# Osteoblastic erythropoietin is not required for bone mass accrual

**DOI:** 10.1093/jbmrpl/ziae052

**Published:** 2024-04-15

**Authors:** Giulia Lanzolla, Christophe Merceron, Mohd Parvez Khan, Elena Sabini, Amato Giaccia, Ernestina Schipani

**Affiliations:** Department of Orthopaedic Surgery, University of Pennsylvania, Perelman School of Medicine, Philadelphia, PA 19104, United States; Department of Orthopaedic Surgery, School of Medicine, University of Michigan, Ann Arbor, MI 48109, United States; Department of Orthopaedic Surgery, University of Pennsylvania, Perelman School of Medicine, Philadelphia, PA 19104, United States; Department of Orthopaedic Surgery, School of Medicine, University of Michigan, Ann Arbor, MI 48109, United States; Department of Orthopaedic Surgery, University of Pennsylvania, Perelman School of Medicine, Philadelphia, PA 19104, United States; Department of Radiation Oncology, Stanford University Medical School, Stanford, CA 94304, United States; Department of Orthopaedic Surgery, University of Pennsylvania, Perelman School of Medicine, Philadelphia, PA 19104, United States; Department of Orthopaedic Surgery, School of Medicine, University of Michigan, Ann Arbor, MI 48109, United States

**Keywords:** erythropoietin, osteoblasts, bone mass accrual, erythropoiesis, bone marrow

## Abstract

Erythropoietin (EPO), primarily produced by interstitial fibroblasts in the kidney during adulthood, and its receptor are well-known for their crucial role in regulating erythropoiesis. Recent research has unveiled an additional function of circulating EPO in the control of bone mass accrual and homeostasis through its receptor, which is expressed in both osteoblasts and osteoclasts. Notably, cells of the osteoblast lineage can produce and secrete functional EPO upon activation of the hypoxia signaling pathway. However, the physiological relevance of osteoblastic EPO remains to be fully elucidated. This study aimed to investigate the potential role of osteoblastic EPO in regulating bone mass accrual and erythropoiesis in young adult mice. To accomplish this, we employed a mutant mouse model lacking EPO specifically in mesenchymal progenitors and their descendants. Our findings indicate that in vivo loss of EPO in the osteoblast lineage does not significantly affect either bone mass accrual or erythropoiesis in young adult mice. Further investigations are necessary to comprehensively understand the potential contribution of EPO produced and secreted by osteoblast cells during aging, repair, and under pathological conditions.

## Graphical Abstract

**Graphical Abstract:**
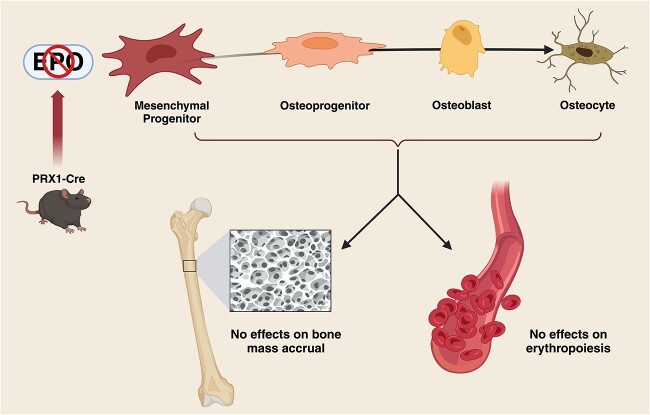

## Introduction

Erythropoietin (EPO) is a hormone that stimulates erythropoiesis through the activation of its receptor (EPOR), present in erythroid progenitor cells. In adults, more than 90% of EPO is produced in the kidney by a subset of peritubular interstitial fibroblasts.^1^ The key regulatory mechanism of EPO expression is hypoxia via the activation of the hypoxia-inducible factor (HIF) pathway.^2^ Anemia stimulates EPO production by inducing local hypoxia. Unlike EPO, EPOR expression is not affected by hypoxia or anemia.^3^EPOR is also expressed in a variety of non-hematopoietic tissues, implying that EPO may have functions beyond the regulation of erythropoiesis.^1^ Emerging research indicates that circulating EPO plays a role in bone mass accrual and homeostasis.^2^^,^^4^ EPORs have been identified in both osteoblasts and osteoclasts.^5^^,^^6^ This distribution suggests that circulating EPO affects bone mass through direct effects on bone cells.^7-10^ Conditional deletion of EPOR in osteoblasts resulted in an increased vertebral bone volume.^5^^,^^6^ Conversely, loss of EPO in osteoclasts did not cause any overt phenotype per se but prevented the bone loss observed upon continuous administration of EPO.^5^^,^^6^ These results indicate that bone loss secondary to EPO treatment occurs through a direct action on both osteoblastic cells and osteoclasts.

Cells of the osteoblast lineage can produce and secrete functional EPO.^11^ Moreover, increased activity of HIF-2 in osteoblastic cells stimulates the expression of osteoblastic EPO and causes polycythemia.^11^ Given the evidence that systemic EPO affects bone mass through its actions on bone cells, in this study, we investigated whether osteoblastic EPO locally contributes to the control of bone mass accrual and erythropoiesis using a mouse model genetically lacking EPO in mesenchymal progenitors and their descendants.

## Material and methods

### Generation of PRX;EPO^f/f^ mutant mice

Generation and genotyping methods of Prx1-Cre (FVB/N), EPO^f/f^ (C57BL/6) transgenic mice have been previously described.^12^^,^^13^ Prx1-Cre transgenic male mice were bred with homozygous EPO^f/f^ females to obtain PRX;EPO^f/+^ male mice. These newly generated males were crossed with EPO^f/f^ females to generate PRX;EPO^f/f^ mutants and PRX;EPO^f/+^ and EPO^f/f^ control littermates.

All procedures involving mice were performed in accordance with the NIH guidelines for the use and care of live animals and were approved by the University of Michigan Institutional Animal Care and Use Committee (IACUC) (Protocol number: PRO00007215) and by the University of Pennsylvania IACUC (Protocol number: 806981**)**.

### Recombination of the floxed EPO locus in bone marrow stromal cells

EPO^f/f^ and PRX-EPO^f/f^ bone marrow stromal cells (BMSCs) were obtained upon flushing of 6-wk-old bones, cultured in high-glucose DMEM supplemented with 15% FBS and 1% Penicillin/Streptomycin, and incubated at 37°C, 5% CO_2_ under a humidified atmosphere. Medium was renewed every 24 h during the first 48 h to remove nonadherent cells; the medium was next changed every 2-3 d. Cells were cultured for 7 d.

The efficiency of deletion was assessed by qPCR for 2-LoxP performed on genomic DNA at the indicated time. Data were normalized to *Von Hippel–Lindau* (*Vhl*) as the internal reference for genomic DNA. Primers used for genotyping are provided in Table 1.

**Table 1:** Genotyping and 2-LoxP primers.

	Sequence	Amplicon (bp)
*EPO*	Fwd 5′-AGTGAAGTTTGGCCGAGAAG-3′	341
	Rev 5′-GTGGGACGTTCTGGAAGAAA-3′	

### Blood analysis

Blood was collected by cardiac puncture under deep anesthesia (surgical plane) using 1 mL syringe fitted with a 23 G needle. Blood samples were split into 2 fractions. The first fraction was collected in K_2_EDTA- coated tubes (BD Microtainer – 365974) for complete blood count (CBC). CBC was performed by the Unit for Laboratory Animal Medicine–In Vivo Animal Core using a Hemavet 950FS (Drew Scientific). The other fractions were collected in serum separator tubes (BD Microtainer–365 967). Blood was allowed to clot for 30 min at room temperature prior to centrifugation for 5 min at 10000 *g*. Samples were stored at −80°C before EPO quantification by ELISA according to the manufacturer’s instructions (RnD Systems-MEP00B).^11^

### MicroCT analysis

Femur length was measured by microCT. Femurs were placed vertically in a tube sample holder for a stable and symmetrical sample positioning, and they were fixed in gauzes to avoid slight movements that may affect scanning at a high resolution. 3D microCT images of the femurs were generated using the eXplore Locus SP system (GE Healthcare). Images were analyzed and quantified using Microview Software (Parallax Innovations). ROI was defined as a percentage of the overall bone length. Both trabecular and cortical ROI lengths were set at 10% of the overall bone length. Trabecular ROI position was defined by having its closest edge located 5% away from the growth plate. Cortical ROI position was defined by having its center at 50% of the total bone length.

### H&E and TRAP stainings

For H&E, and tartrate-resistant acidic phosphatase (TRAP) stainings, femurs were fixed in 4% PFA/PBS, processed, and embedded in paraffin as previously reported.^14^ TRAP staining was performed according to the manufacturer’s indications (Sigma-Aldrich 387A-1KT). H&E and TRAP-stained sections were used for static histomorphometry analysis. All images were acquired with an Eclipse E800 microscope (Nikon).

### Calcein labeling and generation of methylmethacrylate sections

Mice were injected intraperitoneally with calcein (Sigma-C0875) (40 mg/kg^−1^) 10 and 3 d prior to sacrifice. Femurs were collected and fixed in PFA 4% before embedding in methylmethacrylate as previously described.^15^^,^^16^ Sections of 5 μm thickness were generated, cover-slipped without any staining, and used for dynamic histomorphometric analysis. All images were generated using a FITC filter mounted on an Eclipse E800 microscope (Nikon).

### Histomorphometry

Histomorphometric analysis of trabecular bone was performed on paraffin or methylmethacrylate sections of femurs generated as described above. ROI was defined as a percentage of the overall bone length. Trabecular ROI length was set at 10% of the total length of the bone, and its position was defined by having the closest edge located 5% away from the growth plate. Cortical bone was excluded from the ROI. Histomorphometric measurements were performed in a randomized and blind manner using the Bioquant Osteo software V17.2.6 (Bioquant Image Analysis Corp.), according to the standard procedures.^14^

#### Static histomorphometry

H&E sections were used to quantify trabecular bone volume over tissue volume (BV/TV), bone surface (BS), trabecular number (Tb.N), trabecular thickness (Tb.Th.), trabecular spacing (Tb.Sp.), number of osteoblasts over bone surface (N.Ob/BS). TRAP staining allowed the visualization and proper counting of osteoclast number over bone surface (Oc.N./BS).

#### Dynamic histomorphometry

Single-labeled surface (sLS), double-labeled surface (dLS), mineralizing surface/bone surface (MS/BS), mineral apposition rate (MAR), and bone formation rate/bone surface (BFR/BS) were quantified on unstained methylmethacrylate sections.

### Statistical analysis

The in vitro experiments were performed at least in biological and technical triplicates. For in vivo experiments, a minimum of 6 specimens per condition were analyzed. Data are represented as the mean of the replicates ± SD or SEM. Statistical analysis was performed by using GraphPad Prism, version 9.4.1. When appropriate, a 2-tailed, unpaired Student’s *t*-test or a one-way ANOVA with multiple comparisons was performed to analyze statistical differences between groups. *P*-values <.05 were considered statistically significant.

## Results

Since osteoblastic cells have the capability to produce and secrete functional EPO, we investigated whether osteoblastic EPO contributes to the regulation of bone mass and the stimulation of erythropoiesis. To explore those possibilities, we selectively ablated EPO in the mesenchymal progenitors within the limb bud and their descendants. Our analysis focused on the bone phenotype of mutant mice at 15 wk of age. This study aimed to uncover a potential physiological role of EPO in osteoblastic cells and determine if osteoblastic EPO is essential for controlling bone mass accrual or hematopoiesis.

For our experimental model, we utilized Prx1-Cre transgenic mice^12^ crossed with EPO^f/f^ mice^13^ to create PRX-EPO^f/f^ mutants, alongside PRX-EPO^f/+^ and EPO^f/f^ control littermates. qPCR analysis of genomic DNA extracted from BMSCs of both EPO^f/f^ and PRX-EPO^f/f^ mice, following a brief in vitro culture period, confirmed efficient recombination of the floxed alleles in the mutant cells (Figure S1).

### The in vivo deletion of EPO in mesenchymal progenitors and their descendants does not result in macroscopic defects

**Figure 1:**
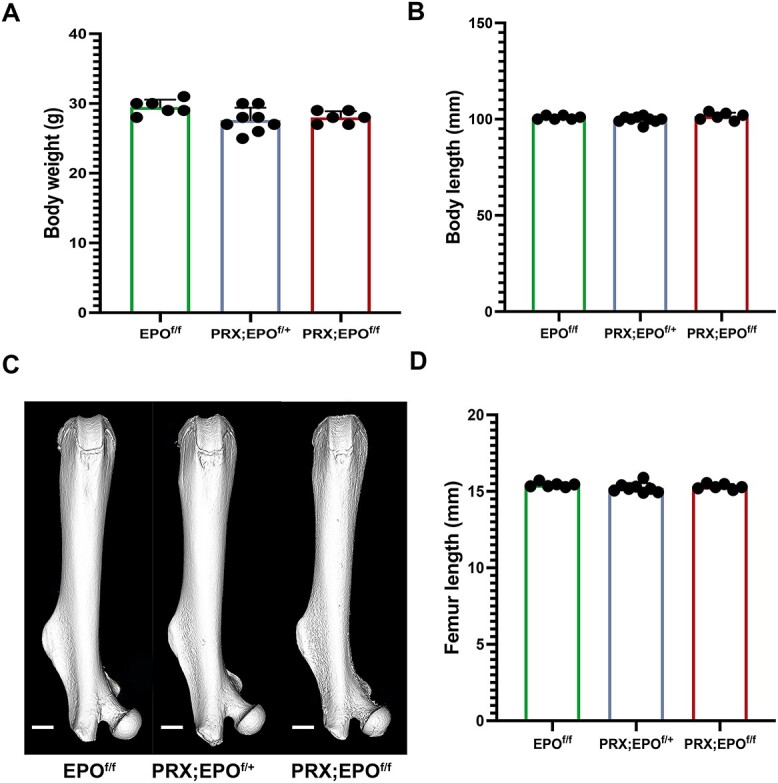
Macroscopic characterization of EPO^f/f^, PRX;EPO^f/+^, and PRX;EPO^f/f^ mice. (A) Body weight of male EPO^f/f^, PRX;EPO^f/+^, and PRX;EPO^f/f^ mice at 15 wk of age. (B) Body length of male EPO^f/f^, PRX;EPO^f/+^, and PRX;EPO^f/f^ mice at the same time point. (C) Representative microCT 3D reconstruction images of male EPO^f/f^, PRX;EPO^f/+^, and PRX;EPO^f/f^ femurs at 15 wk of age. Scale bars=1mm. (D) Femur length of male EPO^f/f^, PRX;EPO^f/+^, and PRX;EPO^f/f^ mice femurs at 15 wk of age. At least, 6 specimens per group were analyzed. *P*-value significance refers to the comparison to EPO^f/f^: *P*>.05 by one-way ANOVA with multiple comparisons (not shown).

Male and female EPO^f/f^, PRX;EPO^f/+^, and PRX;EPO^f/f^ mice at 15 wk post-birth exhibited normal phenotypes, with no detectable patterning defects. Measurements of total body length (from the nose tip to the terminal caudal bone tip) to the nearest 0.5 mm using a clear plastic ruler showed no significant differences between PRX-EPO^f/f^ mutants and PRX;EPO^f/+^ and EPO^f/f^ control littermates in both sexes (Figure 1 and Figure S2A). Body weight, recorded to the nearest 0.1 g, was also comparable across all groups (Figure 1A and Figure S2A). Furthermore, femur length measurements revealed no significant differences across the groups (Figure 1C and Figure S2A).

### The in vivo loss of EPO in these cells does not significantly impact erythropoiesis

**Figure 2:**
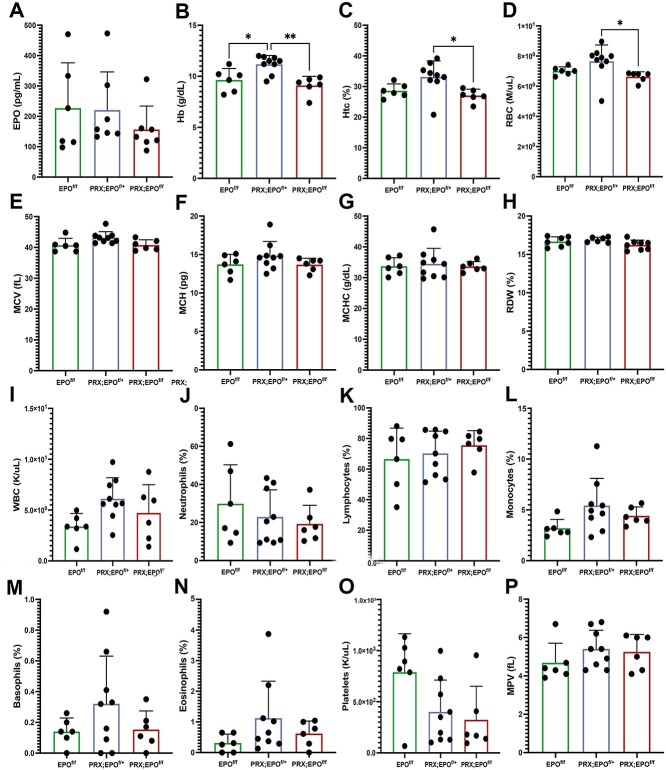
Hematopoiesis and circulating levels of EPO in EPO^f/f^, PRX;EPO^f/+^, and PRX;EPO^f/f^ mice. Measurements of EPO (A), hemoglobin (Hb) (B), hematocrit (Htc) (C), Red blood cells (RBCs) (D), mean corpuscular volume (MCV) (E), mean corpuscular hemoglobin (MCH) (F), mean corpuscular hemoglobin concentration (MCHC) (G), red blood cells distribution width (RDW) (H), white blood cells (WBC) (I), neutrophils (J), lymphocytes (K), monocytes (L), basophils (M), eosinophils (N), platelet (O), mean platelets volume (MPV), (P) in male EPO^f/f^, PRX;EPO^f/+^, and PRX;EPO^f/f^ mice at 15 wk of age. At least, 6 specimens per group were analyzed; *P*-value significance refers to the comparison to the EPO^f/f^ specimens: ^*^P ≤ .05; ^*^^*^P ≤ .01 by one-way ANOVA with multiple comparisons.

Although circulating EPO levels were slightly reduced in both male and female PRX;EPO^f/f^ mice compared to gender-matched EPO^f/f^ controls (Figure 2 and Figure S3), this difference was not statistically significant. Parameters such as hemoglobin (Hb), hematocrit (Htc), and RBC count were similar in PRX;EPO^f/f^ mutants and EPO^f/f^ controls across both genders (Figure 2 and Figure S3).

Interestingly, Hb levels were significantly higher in PRX;EPO^f/+^ males compared to sex-matched EPO^f/f^ and PRX;EPO^f/f^, while Htc and RBC were significantly elevated in PRX;EPO^f/+^ when compared to PRX;EPO^f/f^ (Figure 2). In females, these parameters were significantly increased in PRX;EPO^f/+^ compared to both EPO^f/f^ and PRX;EPO^f/f^ littermates (Figure S3). The physiological implications of these findings remain unclear.

Other erythrocyte indices, including mean corpuscular volume, mean corpuscular hemoglobin, mean corpuscular hemoglobin concentration, and red blood cells distribution width, showed no significant variances across groups, regardless of gender (Figure 2 and Figure S3).

Furthermore, there was no significant difference in the count of circulating white blood cells, neutrophils, lymphocytes, monocytes, eosinophils, basophils, and platelets in both male and female PRX;EPO^f/f^ mutant mice compared to EPO^f/f^ and PRX;EPO^f/+^ littermates (Figure 2 and Figure S3).

### The in vivo loss of EPO in mesenchymal progenitors and their descendants does not affect bone mass accrual in either trabecular or cortical bone

**Figure 3:**
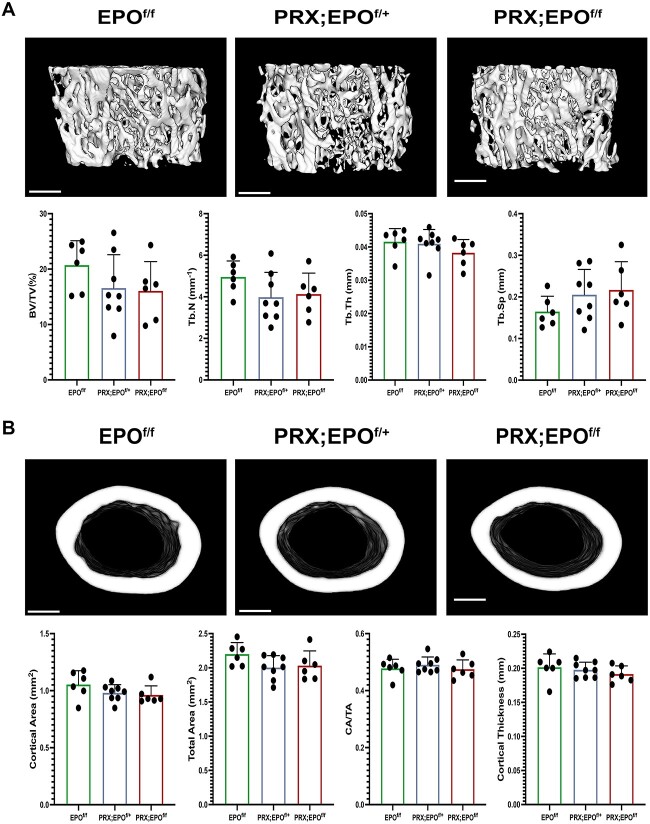
MicroCT analysis of trabecular bone in distal metaphysis and cortical bone in mid-diaphysis of femurs isolated from 15-wk-old male EPO^f/f^, PRX;EPO^f/+^, and PRX;EPO^f/f^ mice. (A) Representative images of trabecular bone are shown at the top. At the bottom, quantification of Bone Volume/Tissue Volume (BV/TV), Tb.N, Tb.Th, and Trabecular Separation (Tb.Sp) is provided. (B) Representative images of cortical bone are shown at the top. At the bottom, the quantification of Cortical Area, Total Area, Cortical Area/Total Area (CA/TA), and Cortical Thickness is provided. At least, 6 specimens per group were analyzed. Scale bars: 100 μm. *P*-value significance refers to the comparison to EPO^f/f^: *P*>.05 by one-way ANOVA with multiple comparisons (not shown).

MicroCT analysis of femurs showed no significant differences in trabecular or cortical bone mass in PRX;EPO^f/f^ mutants compared to EPO^f/f^ controls in both sexes (Figure 3 and Figure S2C and D). Specific parameters, such as trabecular BV/TV, Tb.N, and Tb.Th, were slightly lower in PRX-EPO^f/f^ male bones compared to EPO^f/f^ controls, while trabecular separation (Tb.S) was higher in mutants (Figure 3). However, none of these differences reached statistical significance. Additionally, cortical parameters like cortical area/total area (CA/TA) and cortical thickness (C.Th.) showed no differences between mutant and control specimens, regardless of sex (Figure 3 and Figure S2C).

**Figure 4:**
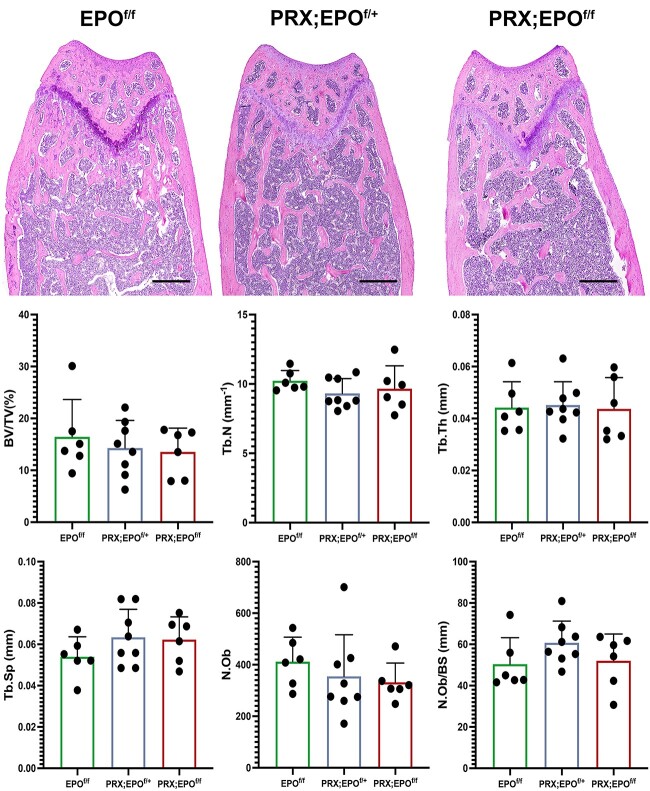
Histological analysis of the EPO^f/f^, PRX;EPO^f/+^, and PRX;EPO^f/f^ distal femur. Representative paraffin sections of distal femur metaphysis isolated from 15-wk-old male EPO^f/f^, PRX;EPO^f/+^, and PRX;EPO^f/f^ mice stained with H&E are shown at the top. At the bottom, the quantification of Bone Volume/Tissue Volume (BV/TV), Tb.N, Tb.Th, Trabecular Separation (Tb.Sp), Osteoblast Number (N.Ob), and Osteoblast Number/Bone surface (N.Ob/BS) is provided. Scale bars=200 μm. At least, 6 specimens per group were analyzed; *P*-value significance refers to the comparison to EPO^f/f^: *P* > .05 by one-way ANOVA with multiple comparisons (not shown).

**Figure 5:**
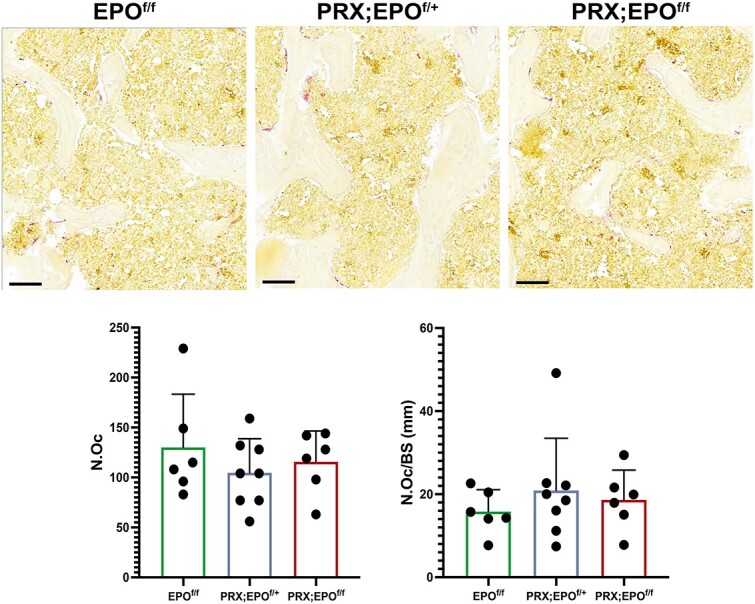
TRAP staining of histological sections of the distal metaphysis of femurs isolated from 15-wk-old male EPO^f/f^, PRX;EPO^f/+^, and PRX;EPO^f/f^ mice. Representative images are shown at the top. At the bottom, the quantification of Osteoclast Number (Oc.N) and Osteoclast Number/Bone Surface (Oc.N/BS) is provided. Scale bars=200 μm. At least, 6 specimens per group were analyzed. *P*-value significance refers to the comparison to EPO^f/f^: *P*>.05 by one-way ANOVA with multiple comparisons (not shown).

**Figure 6:**
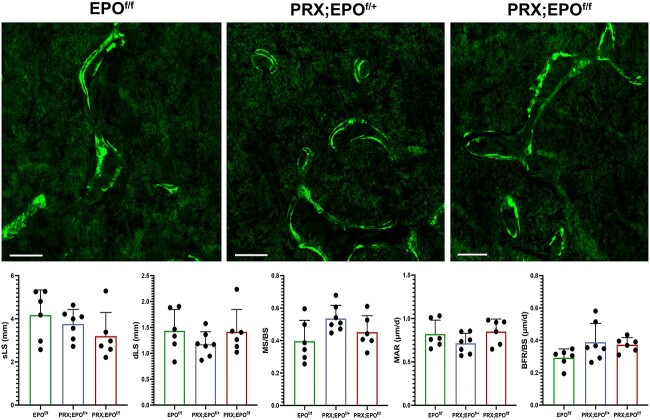
Dynamic histomorphometric analysis of distal femurs isolated from 15-wk-old male EPO^f/f^, PRX;EPO^f/+^, and PRX;EPO^f/f^ mice. Representative double-calcein labeling images are shown at the top. At the bottom, the quantification of sLS, dLS, MS/BS, MAR, BFR/BS. Scale bars=200 μm. At least, 6 specimens per group were analyzed. *P*-value significance refers to the comparison to EPO^f/f^: *P* > .05 by one-way ANOVA with multiple comparisons (not shown).

Given the absence of major gender-based differences in macroscopic characterization and microCT analysis, histomorphometry was conducted only in males. Static histomorphometric analysis of trabecular bone corroborated the microCT findings (Figure 4). The number of osteoblasts (N.Ob), even upon correction over the bone surface (N.Ob/BS), was similar in mutant and control bones (Figure 4), and no difference was observed in the number of osteoclasts over bone surface (N.Oc/BS) between PRX-EPO^f/f^ male bones and EPO^f/f^ controls (Figure 5). Additionally, the sLS, dLS, MS/BS, MAR, and BFR/BS were comparable in PRX-EPO^f/f^ males and EPO^f/f^ littermate controls (Figure 6).

Lastly, analysis of all those parameters showed that PRX;EPO^f/+^ mice were virtually indistinguishable from PRX;EPO^f/f^ mutant mice and EPO^f/f^ controls.

Our findings indicate that EPO does not play a significant physiological role as a regulator of bone mass in both trabecular and cortical bone, regardless of sex, in young adult mice.

## Discussion

Although it has long been understood that the kidney is the primary source of EPO in adults, recent research has begun to spotlight the skeleton—specifically, osteoblastic cells—as an additional site for EPO synthesis and release.^11^ Despite this, the physiological impact of EPO production by osteoblastic cells, particularly in relation to hematopoiesis and bone mass accrual, remains poorly understood. This study aimed to delineate the role of osteoblast-derived EPO in bone mass regulation. Moreover, since previous findings had suggested a correlation between reduced expression of EPO in osteoblastic cells at birth and a decrease in bone marrow erythroid progenitors,^11^ we further probed the effects of locally produced EPO by osteoblastic cells on hematopoiesis.

Our research demonstrates that the targeted ablation of the EPO gene in mesenchymal progenitors and their subsequent lineages does not thwart the accumulation of bone mass in young adult mice, within either the trabecular or cortical structures, and does not negatively impinge on erythropoiesis. These findings suggest that the presumed autocrine and paracrine actions of EPO produced by cells within the osteoblast lineage are not significant modulators of the bone mass or erythropoiesis under normal physiological states. However, given that bone characteristics are known to change with age,^17^ additional research is needed to fully understand the role of osteoblastic EPO in bone and bone marrow physiology throughout the lifespan.

Additionally, EPO mRNA presence has been detected in growth plate chondrocytes,^18^ adding another layer of complexity to its role in skeletal biology. Our findings indicate that limb length in PRX-EPO^f/f^ mutant mice mirrors that of controls, suggesting that EPO’s role in the mesenchymal progenitors of the limb bud is not crucial for growth plate development, regardless of sex. Yet, the potential modulatory effect of EPO in the context of pathological conditions, like fracture repair, cannot be excluded. Along those lines, EPOR has been identified in the cartilaginous callus during bone repair stages,^19^ and several studies have suggested a possible therapeutic role of EPO in enhancing bone healing, particularly via promoting endochondral ossification.^20-22^

In our study, using a mouse model with conditional EPO gene deletion in the osteoblastic lineage of limbs, we observed no significant differences in systemic EPO levels compared to control mice. However, this localized deletion means we cannot rule out the possibility that a more widespread disruption of EPO throughout the skeletal system might impact circulating EPO levels.

Moreover, renal insufficiency results in reduced EPO production due to the kidneys’ diminished capacity for hypoxia detection and EPO synthesis, leading to anemia, a frequent complication in chronic kidney disease. Our previous discovery that osteoblastic cells can produce EPO offers an alternative pathway for EPO stimulation in these patients. Consequently, manipulating the hypoxia signaling pathway in osteoblasts to boost EPO production may hold promise, especially in cases of renal insufficiency where traditional renal EPO production is compromised. The potential for using EPO in disease states, including the possibility of pharmacologically enhanced osteoblastic EPO to offset renal insufficiency, deserves further exploration.

In summary, our study emphasizes that the presumed paracrine or autocrine effects of osteoblast-derived EPO do not play a crucial role in regulating bone mass in trabecular or cortical areas, nor are they essential for erythropoiesis in young adult mice. Nonetheless, the significance of osteoblastic EPO in aging, bone regeneration, and its therapeutic potential, particularly in treating anemia associated with chronic kidney disease, warrants additional investigation.

## Supplementary Material

Suppl_figure_ziae052

## Data Availability

The data underlying this article are available in the article and in its online supplementary material.
