# Supplementary material for: Osteoblastic erythropoietin is not required for bone mass accrual
Source: JBMR Plus. 2024 Apr 15;8(6):ziae052. doi: 10.1093/jbmrpl/ziae052 (PMC11102573; doi:10.1093/jbmrpl/ziae052)
Supplement: Suppl_figure_ziae052 [file suppl_figure_ziae052.docx]

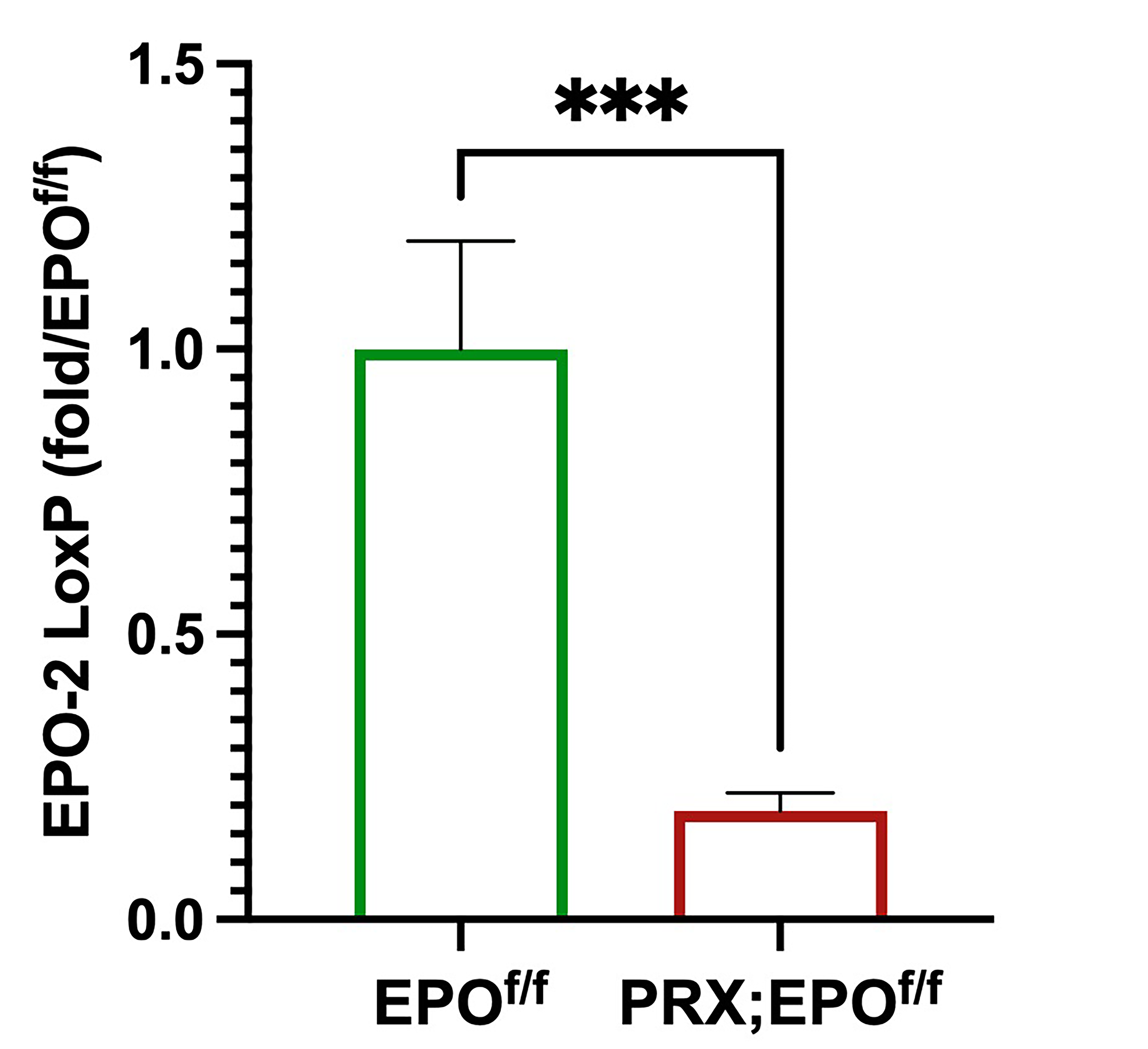


**Supplementary Figure 1:** **Recombination of the floxed *EPO* locus in PRX;EPO^fl/fl^ bone marrow stromal cells.** 2-LoxP qPCR was performed on genomic DNA extracted from EPO^f/f^ and PRX;EPO^f/f^ bone marrow stromal cells upon a brief *in vitro* culture. Data were normalized to *Von Hippel-Lindau* (*Vhl)* as internal reference for genomic DNA. P-value significance refers to the comparison to EPO^f/f^: *** P ≤ 0.001 by student’s t-test.


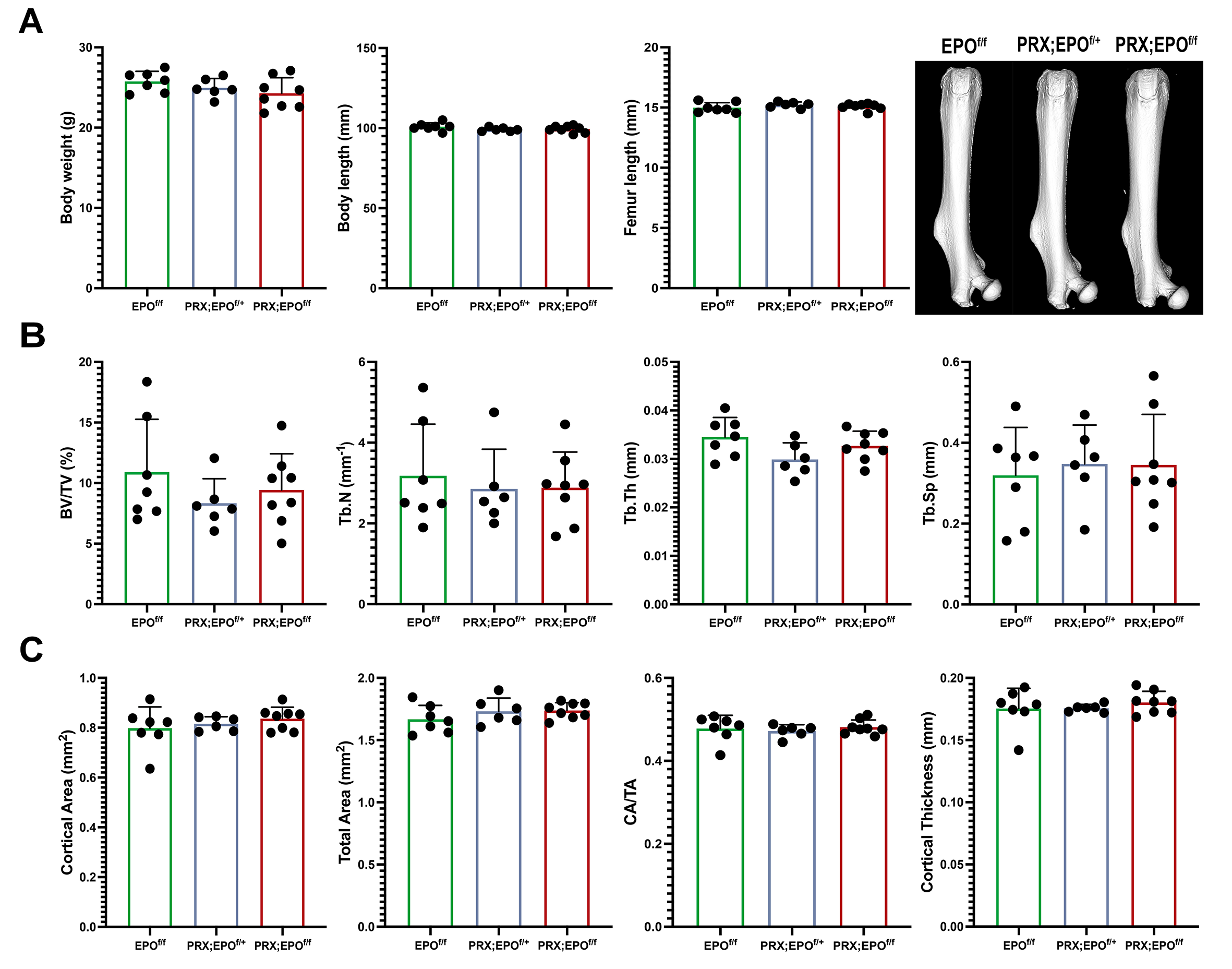


**Supplementary Figure 2: Macroscopic characterization of EPO^f/f^, PRX;EPO^f/+^ and PRX;EPO^f/f^ female mice.**

(A) Body weight, body length and femur length of female EPO^f/f^, PRX;EPO^f/+^ and PRX;EPO^f/f^ mice at 15 weeks of age. Representative micro-CT 3D reconstruction images are shown on the right. Scale bars=1mm. (B) MicroCT analysis of trabecular bone in distal metaphysis and (C) cortical bone in mid-diaphysis of femurs isolated from 15-week-old female EPO^f/f^, PRX;EPO^f/+^ and PRX;EPO^f/f^ mice. The quantification of Bone Volume/Tissue Volume (BV/TV), Trabecular Number (Tb.N), Trabecular Thickness (Tb.Th), Trabecular Separation (Tb.Sp), Cortical Area, Total Area, Cortical Area/Total Area (CA/TA) and Cortical Thickness is provided. Scale bars=100 μm. At least, 6 specimens per group were analyzed. P-value significance refers to the comparison to EPO^f/f^: P>0.05 by one-way analysis of variance (ANOVA) with multiple comparisons (not shown).


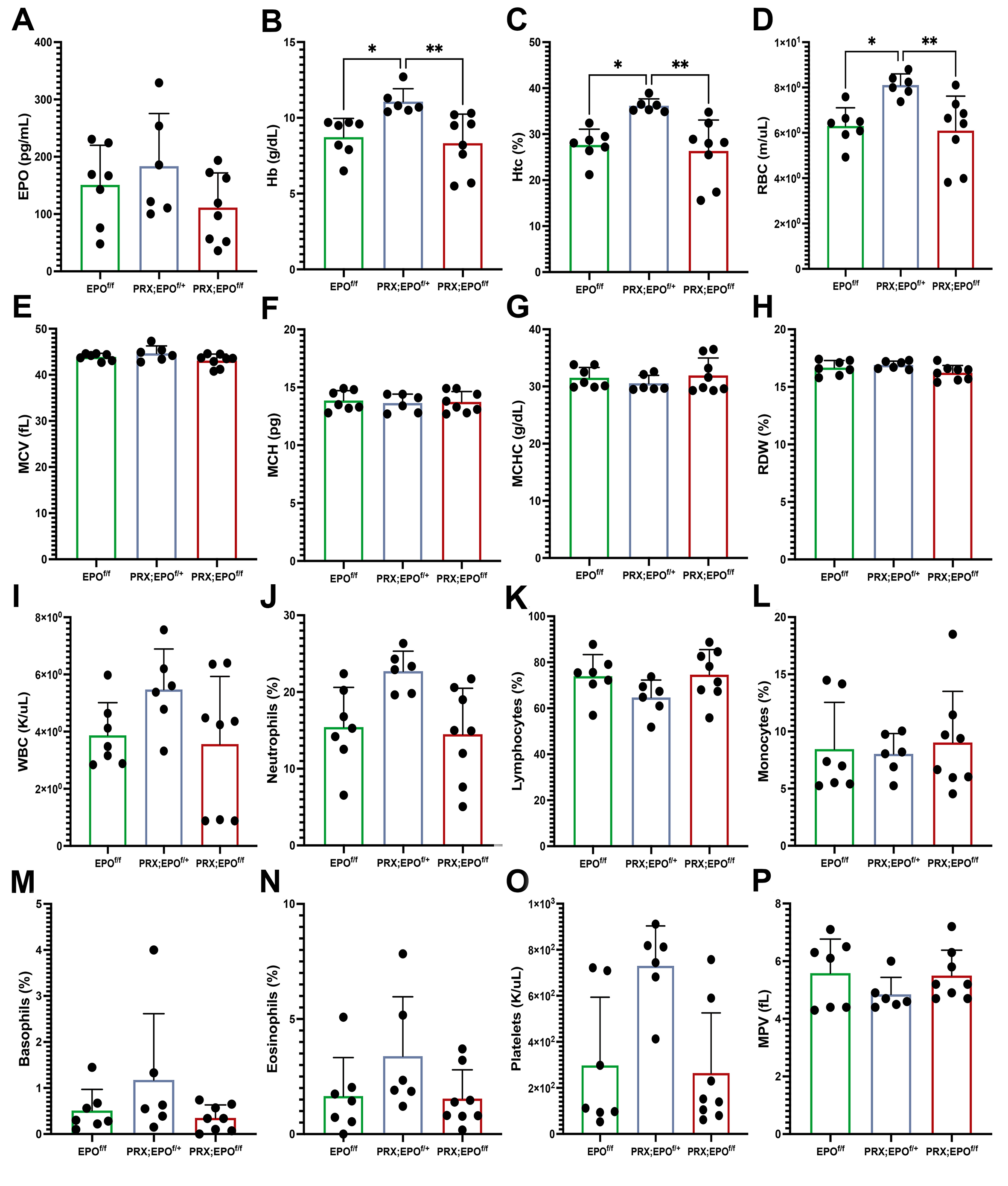


**Supplementary Figure 3: Hematopoiesis and circulating levels of erythropoietin in EPO^f/f^, PRX;EPO^f/+^ and PRX;EPO^f/f^ mice.**

Measurements of erythropoietin (EPO) (A), hemoglobin (Hb) (B), hematocrit (Htc) (C), Red blood cells (RBC) (D), Mean Corpuscular Volume (MCV) (E), Mean Corpuscular Hemoglobin (MCH) (F), Mean Corpuscular Hemoglobin Concentration (MCHC) (G), Red Blood Cells Distribution Width (RDW) (H), White Blood Cells (WBC) (I), neutrophils (L), lymphocytes (M), monocytes (N), basophils (O), eosinophils (P), Platelet (Q), Mean Platelets Volume (MPV) (R) in female EPO^f/f^, PRX;EPO^f/+^ and PRX;EPO^f/f^ mice at 15 weeks of age. At least, 6 specimens per group were analyzed. P-value significance refers to the comparison to the EPO^f/f^ specimens: * P ≤ 0.05; ** P ≤ 0.01 by one-way analysis of variance (ANOVA) with multiple comparisons.
